# Relationship of aortic stiffness measured by cardiovascular magnetic resonance to arterial stiffness estimates by tonometry

**DOI:** 10.1186/1532-429X-11-S1-P230

**Published:** 2009-01-28

**Authors:** Ilias Kylintireas, Colin Cunnington, Malcolm Kohler, Jonathan Diesch, Corinne Trevitt, Robin Choudhury, Steffen Petersen, Stefan Neubauer, Matthew Robson, Paul Leeson

**Affiliations:** University Of Oxford, Oxford, UK

**Keywords:** Cardiovascular Magnetic Resonance, Arterial Stiffness, Pulse Wave Velocity, Aortic Stiffness, Cardiovascular Magnetic Resonance Imaging

## Introduction

Arterial stiffness assessed by arterial tonometry – in particular pulse wave velocity and augmentation index – is associated with increased cardiovascular risk. It is now possible to use cardiovascular magnetic resonance imaging (CMR) for direct imaging of aortic function. This provides a precise assessment of the elastic properties of the aorta within distinct regions of the aorta. We therefore studied what parameters of aortic function are related to arterial tonometry-derived indices of arterial stiffness.

## Purpose

We compared aortic stiffness assessed by cardiovascular magnetic resonance (CMR) pulse wave velocity (PWV) based on flow, velocity or acceleration, and aortic distensibility (AD)) to widely used arterial stiffness estimates provided by arterial tonometry (pulse wave velocity (PWVS) and pulse wave analysis (AI)).

## Methods

Thirty young healthy volunteers (aged 23–33) underwent tonometry (Sphygmacor) for determination of carotid to femoral PWVS and radial pulse wave analysis for AI estimation. They then underwent CMR to assess aortic elastic properties.

Aortic distensibility was assessed from breath-hold ECG-gated, steady state free precession (SSFP) images (TR = 2.8 ms, echo time, TE = 1.4, 2 mm × 2 mm, slice thickness 7 mm, temporal resolution 40 ms) acquired at the level of the right pulmonary artery through the ascending and proximal descending aorta and through the distal aorta below the diaphragm to determine aortic distensability. Distensibility was calculated as the relative change in area divided by the central pulse pressure.

**Figure 1:**
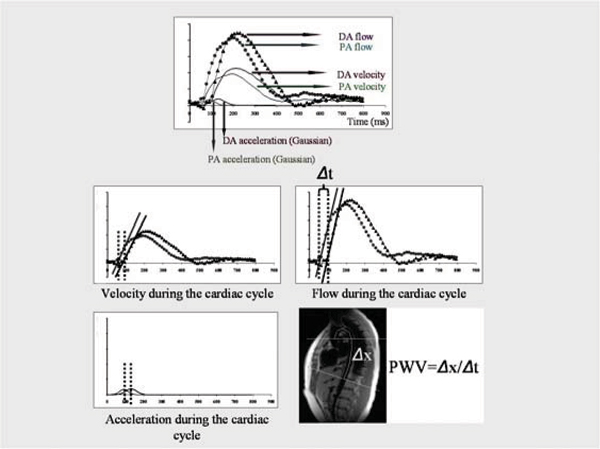
Figure 1

Pulse wave velocity was measured by an ECG-gated, free breathing, spoiled gradient echo phase-encoded acquisition (TR = 1 RRinterval, TE = 2.8 ms, 1.3 mm × 1.3 mm, slice thickness = 5 mm, temporal resolution = 10 ms) at the same locations as the aortic cine images. Three variations of the transit time method were used for calculation of pulse wave velocity (PWV). Arrival of the pulse wave was defined as a) the foot of the curve of mean blood velocity of the blood during the cardiac cycle b) the foot of the curve of total blood flow during the cardiac cycle c) maximum blood acceleration rate (maximum differential of the mean velocity) during the cardiac cycle (Figure 1).

## Results

**Figure 2:**
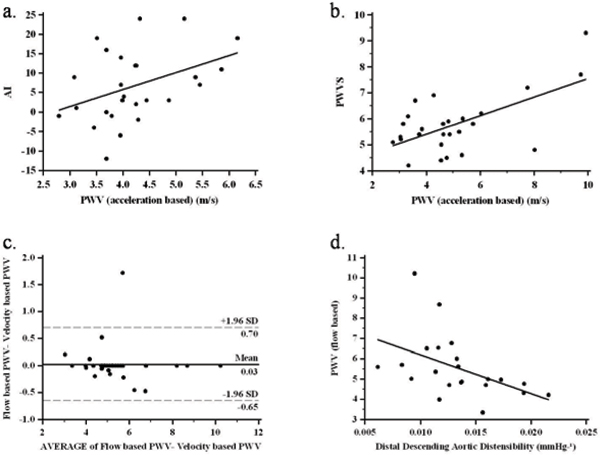
Figure 2

AI was most strongly correlated with acceleration based MRI PWV (r = 0.4012, P < 0.05) (Figure 2a) but with no other measures of PWV or aortic distensibility. PWVS was also most strongly correlated with acceleration based MRI PWV over the whole length of the aorta (r = 0.615, P < 0.001) (Figure 2b) but also related to flow (r = 0.484 P < 0.05) and velocity (r = 0.372, P < 0.05) based MRI PWV measurements.

Aortic pulse wave velocity calculated by all three MR methods yielded an inverse correlation with descending aortic distensibility (r = -0.521, P < 0.05) (Figure 2d) but PWVS and AI did not correlate with aortic distensibility

## Conclusion

PWVS and AI most closely reflect acceleration-based CMR PWV measurements of overall aortic stiffness. Arterial tonometry has been validated in large scale clinical studies suggesting this CMR method may be most useful for interpretation of clinical risk. Regional and overall aortic measures of elasticity were not strongly related. However, CMR PWV measures inversely correlated with aortic distensibility, suggesting that MR PWV values may provide a closer estimate of local aortic elastic properties.

